# CsPRMT5-mediated histone H4R3 dimethylation negatively regulates resistance to gray blight in tea plants (*Camellia sinensis* L.)

**DOI:** 10.1093/hr/uhaf100

**Published:** 2025-04-09

**Authors:** Huanyun Peng, Yan Wang, Biying Zhu, Yuanrong Wang, Mengxue Han, Shupei Zhang, Tianyuan Yang, Fei Wang, Zhaoliang Zhang

**Affiliations:** National Key Laboratory for Tea Plant Germplasm Innovation and Resource Utilization, Anhui Agricultural University, West 130 Changjiang Road, Hefei, Anhui 230036, China; National Key Laboratory for Tea Plant Germplasm Innovation and Resource Utilization, Anhui Agricultural University, West 130 Changjiang Road, Hefei, Anhui 230036, China; National Key Laboratory for Tea Plant Germplasm Innovation and Resource Utilization, Anhui Agricultural University, West 130 Changjiang Road, Hefei, Anhui 230036, China; National Key Laboratory for Tea Plant Germplasm Innovation and Resource Utilization, Anhui Agricultural University, West 130 Changjiang Road, Hefei, Anhui 230036, China; National Key Laboratory for Tea Plant Germplasm Innovation and Resource Utilization, Anhui Agricultural University, West 130 Changjiang Road, Hefei, Anhui 230036, China; National Key Laboratory for Tea Plant Germplasm Innovation and Resource Utilization, Anhui Agricultural University, West 130 Changjiang Road, Hefei, Anhui 230036, China; National Key Laboratory for Tea Plant Germplasm Innovation and Resource Utilization, Anhui Agricultural University, West 130 Changjiang Road, Hefei, Anhui 230036, China; National Key Laboratory for Tea Plant Germplasm Innovation and Resource Utilization, Anhui Agricultural University, West 130 Changjiang Road, Hefei, Anhui 230036, China; National Key Laboratory for Tea Plant Germplasm Innovation and Resource Utilization, Anhui Agricultural University, West 130 Changjiang Road, Hefei, Anhui 230036, China

## Abstract

Gray blight is a serious foliar disease that significantly threatens tea plant cultivation. Although dynamic histone methylation was reported in regulating plant immunity, the specific roles of this epigenetic modification in tea plant disease resistance have yet to be fully elucidated. This study demonstrates that the protein arginine methyltransferase CsPRMT5, which catalyzes the symmetric dimethylation of histone H4R3 (H4R3sme2), is involved in the tea plant response to gray blight. Transcription of *CsPRMT5* and the level of histone H4R3 methylation in tea were downregulated following infection by the fungal pathogen *Pseudopestalotiopsis* (*Ps*). A negative correlation was observed between the resistance of tea plants to *Ps* and the expression level of *CsPRMT5* across various cultivars. Downregulation of *CsPRMT5* expression led to reduced H4R3sme2 levels, elevated expression of defense-related genes, and lower reactive oxygen species (ROS) production after *Ps* infection, thus enhancing pathogen resistance of tea. Furthermore, complementation of *Atprmt5* mutant with *CsPRMT5* restored the susceptibility to *Ps* infection in *Arabidopsis*. Chromatin Immunoprecipitation Sequencing (ChIP-seq)and Chromatin Immunoprecipitation quantitative PCR (ChIP-qPCR) analyses revealed that CsPRMT5 binds to defense-related genes, including *CsMAPK3*, and regulates their expression through H4R3sme2 modification. Collectively, the results indicate that CsPRMT5 negatively regulates the immune response to pathogens through repressing *CsMAPK3* expression in tea plants.

## Introduction

Tea, a nonalcoholic beverage appreciated globally, provides substantial economic, health, and cultural advantages, establishing it as a fundamental aspect of daily life for millions [1, 2]. However, tea plants encounter numerous biotic and abiotic stresses [3]. Gray blight is one of the most destructive fungal leaf diseases, inflicting severe damage on tea leaves and leading to considerable economic losses [4–6]. To date, little is known about the molecular mechanisms by which tea plants adapt to the infection of *Ps*, which limits the further design of molecular breeding.

Recent research has highlighted the molecular mechanisms underlying tea plant defense against gray blight disease, emphasizing the critical roles of oxidative bursts and hormones in modulating plant immune responses [6–8]. After *Ps* infection, the expression of essential genes related to phenylpropanoid and flavonoid production increase, including phenylalanine ammonia-lyase (PAL), while genes associated with photosynthesis decrease concurrently in tea plants [6, 9]. Additionally, the transcription of the defensive response genes is tightly regulated by numerous transcription factors, including WRKY and NAC [9–11]. This intricate reprogramming of gene expression necessitates a swift and highly coordinated response at both the transcriptional and post-transcriptional levels.

Increasing evidence suggests that epigenetic processes, including DNA methylation and histone modification, play an essential role in the regulation of transcription, significantly influencing plant immune responses. Histone methylation, a prevalent and critical epigenetic modification, occurs on both lysine and arginine residues. Emerging findings indicate that histone lysine methylation marks play essential roles in plant responses to stresses [12–14]. For instance, JMJ27 activates defense genes like *PR1* and *PR3*, while also repressing negative regulators of defense genes by modulating H3K9 methylation [13]. Similarly, the H3K4 demethylase JMJ14 regulates immunological responses by regulating the deposition of H3K4me3 marks, which in turn affects the transcription of key defense genes, including *PR1*, *FMO1*, and *SNI1* [14]. Additionally, OsJMJ705 removes H3K27me3 from defense-related genes, thereby enhancing resistance [15].

In eukaryotes, histone arginine methylation is a widespread and essential post-translational modification crucial for various biological activities [16, 17]. It is categorized into three types based on the modification form: monomethyl arginine (MMA), asymmetric dimethyl arginine (ADMA), and symmetric dimethyl arginine (SDMA) [16]. The enzymes that catalyze this modification, known as protein arginine methyltransferases (PRMTs), are classified into four groups based on substrate specificity [18]. Type I PRMTs (PRMT1, PRMT3, PRMT4/CARM, PRMT6, and PRMT8) catalyze the formation of ADMA at H3R2/H4R3 sites, activating transcription and ribosomal synthesis, with PRMT1 playing a crucial role in early mouse development. Type II (PRMT5, PRMT9), which mediates the deposition of SDMA at H3R8/H4R3 sites, represses gene expression. Type III (mainly PRMT7), which only generates MMA, and Type IV, which specifically methylates secondary amine groups on arginine residues (a process found only in yeast) [19–21]. The function of PRMTs is highly conserved across evolution, and abnormal expression is closely associated with developmental defects and cancer in mammals.

Recently, genetic studies in model plant *Arabidopsis thaliana* have shown that mutations in AtPRMT3, AtPRMT4a/4b, AtPRMT5, AtPRMT6, and AtPRMT10 lead to significant phenotypic abnormalities (such as delayed flowering and root development defects), confirming the universal regulatory role of arginine methylation in growth and development [22–25]. Among these, PRMT5 (AtSKB1) serves as a core regulatory factor by catalyzing the symmetric dimethylation of histone H4R3 (H4R3sme2), which inhibits transcription of target genes and is involved in plant growth and development and abiotic [25–31]. Additionally, PRMT5 can dynamically modify non-histone substrates (such as LSM4 and AGO2), thereby antagonistically regulating plant responses to both biotic and abiotic stresses [32–34].

Furthermore, recent studies have demonstrated that PRMT5 is involved in plant immunity across multiple species [25]. In Arabidopsis, AtPRMT5 functions as a negative regulator of the immune response to oomycetes and *AvrRpt* pathogens [35, 36]. Specifically, bacterial infection leads to a decrease in PRMT5 expression, accompanied by reduced arginine methylation of key proteins such as AGO2 and LSM4 [33–35]. Through its dual regulation of AtAGO2, PRMT5 modulates the plant’s immune response to pathogens. In contrast, the role of OsPRMT5 in rice (*Oryza sativa*) resistance to blast disease is distinct. Research indicates that OsPRMT5 acts as a positive regulator of immunity in rice. Upon infection with the rice blast fungus, OsPRMT5 regulates AGO2 activity by arginine methylation and interacts with miR1875 to influence the rice immune response [33]. The results suggest that PRMT5 may have varied modes of action among different species, and its role in the disease resistance mechanisms of tea plants requires further investigation.

**Figure 1:**
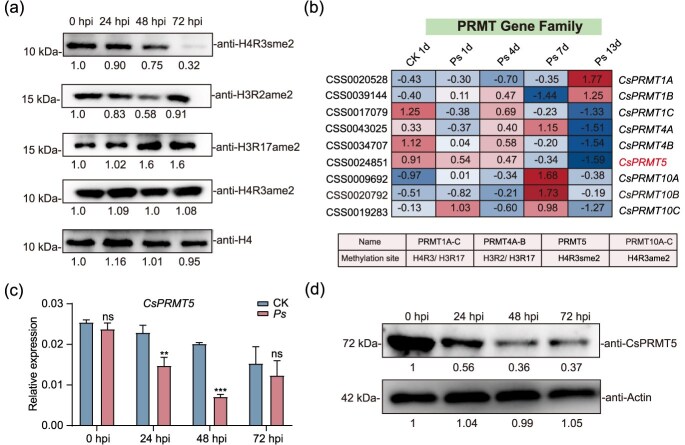
**CsPRMT5 is repressed by the pathogen *Pseudopestalotiopsis* (*Ps*) infection**. (a) Histone methylation levels in tea plant leaves following different periods of *Ps* infection. Histone proteins were extracted at 0, 24, 48, and 72 hpi. Numbers below the blots indicate the quantified relative band intensities of the corresponding methylation site. (b) The expression pattern of *PRMT* family genes. FPKM values are represented by a heatmap. (c) Relative expression of CsPRMT5 in leaves after *Ps* infection. Data were denoted as means ± SD (*n* = 3). Statistical significance (*P* < 0.05) was marked with distinct letters, based on Duncan’s multiple range test. (d) Immunoblot analysis of CsPRMT5 protein levels after *P*s infection. Numbers below the blots indicate the quantified relative band intensities, and the value at time 0 was set to 1.

This research investigates the function of CsPRMT5 in response to *Ps* infection in tea plants. Our results demonstrate that *CsPRMT5*-silenced plants upregulated several immune stress response genes, including *CsWRKY40*, *CsNAC*, and *CsMAPK3*, to enhance plant resistance to the gray blight pathogen. Conversely, the overexpression of *CsPRMT5* increased the vulnerability of tea plants to *Ps*. Following *Ps* infection, H4R3sme2 levels decrease as CsPRMT5 dissociates from the chromatin, thereby facilitating the activation of *CsMAPK3* expression. The findings demonstrate that CsPRMT5 functions as a negative regulator of the defense response in tea plants against gray blight, offering new insights into the role of histone arginine methylation in plant immunity.

## Results

### The histone arginine methylation dynamics in response to gray blight

Although many studies have demonstrated that histone lysine methylation contributes to plant disease resistance, the dynamic changes in arginine methylation levels after pathogen infection remain poorly understood [12]. Consequently, we evaluated the histone dimethylation levels at many arginine sites following *Ps* infection at 0, 24, 48, and 72 h postinoculation (hpi). Western blot analysis was performed using specific antibodies against H4R3sme2, H4R3ame2, H3R2ame2, and H3R17ame2. As shown in Fig. 1a, the methylation levels at distinct arginine sites fluctuated after varying durations of *Ps* inoculation. We observed a significant decrease in H3R2ame2 levels at 48 h, followed by a gradual recovery at 72 h. Conversely, the level of H3R17ame2 significantly increased at both 48 and 72 h. The methylation level at the H4R3 site remained unchanged, while the level of H4R3sme2 significantly decreased over time. These findings indicate that plant disease responses are complex processes, involving the methylation of several arginine residues on histones. Various histone methylation alterations may serve unique functions in modulating this response.

To identify *PRMT* genes associated with plant defense response through histone arginine methylation regulation, we performed a genome-wide analysis of the tea *PRMT* family based on their similarity to *AtSKB1* (Table S1). We identified a total of nine PRMT genes. Among these, eight genes were classified into Type I (*PRMT1A-C*, *PRMT4A-B*, *PRMT10A-C*), which catalyze the asymmetric dimethylation of histone H3 at R2 and R17, and histone H4 at R3. One gene was classified as Type II (*PRMT5*), which catalyzes the symmetric dimethylation of histone H4 at R3 (Fig. 1b). Interestingly, the expression pattern of *PRMT5* in the transcriptomic data shows a consistent decline trend over time with the level of H4R3sme2 after pathogen infection (Fig. 1b). Subsequently, we examined the transcript and protein levels of CsPRMT5 in tea plants after *Ps* infection. A significant reduction in CsPRMT5 transcript and protein levels was also observed at 24 and 48 hpi (Fig. 1c and d). These findings indicate that the expression of *CsPRMT5* was inhibited after *Ps* infection. Previous research has demonstrated that AtPRMT5 contributes to plant immunity by catalyzing the arginine methylation of AtAGO2 and LSM4 [33]. Based on these findings, we chose CsPRMT5 in response to gray blight infection of tea plant for further study.

### The correlation between disease resistance and CsPRMT5 expression in different tea plant cultivars

**Figure 2:**
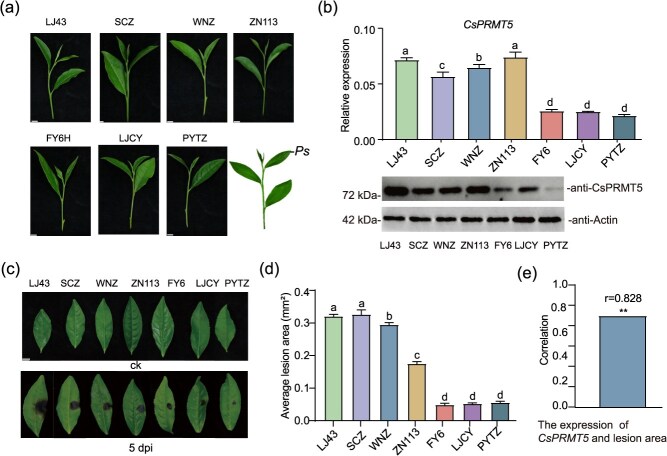
**Disease resistance of tea plants was negatively correlated with the expression of CsPRMT5**. (a) Phenotypes of different tea plant varieties. Scale bars = 1 cm. Inoculate the second leaf with the pathogen (*Ps*). (b) Transcript and protein levels of CsPRMT5 in different tea plant varieties. Data were denoted as means ± SD (*n* = 3); statistical significance (*P* < 0.05) was marked with distinct letters, based on Duncan’s multiple range test. (c) Phenotypes of different tea plant varieties at 5 dpi. (d) Total area of lesions in different tea plant varieties after infection. (e) Correlation between disease resistance and expression level of *CsPRMT5* in different varieties using Pearson’s correlation, ^**^P < 0.01.

To investigate the correlation between *CsPRMT5* expression levels and disease resistance in different tea cultivars, we randomly selected the following seven cultivars for disease resistance experiments (Fig. 2a). The second leaf was selected for pathogen inoculation, and the expression of *CsPRMT5* in unaffected leaves of each cultivar was quantified. Lesion areas were measured 5 days postinoculation (dpi) (Fig. 2b and d). The results showed that the varieties FY6 and PYTZ had relatively low *CsPRMT5* expression levels and exhibited high disease resistance (Fig. 2b and c). Conversely, the varieties SCZ and WNZ had higher *CsPRMT5* transcript levels and showed lower disease resistance (Fig. 2b and c). The lesion area and *CsPRMT5* expression levels were found to be positively correlated across different tea cultivars significantly (r = 0.828, ^**^P < 0.01) (Fig. 2e).

### CsPRMT5 is a negative regulator of gray blight resistance

**Figure 3:**
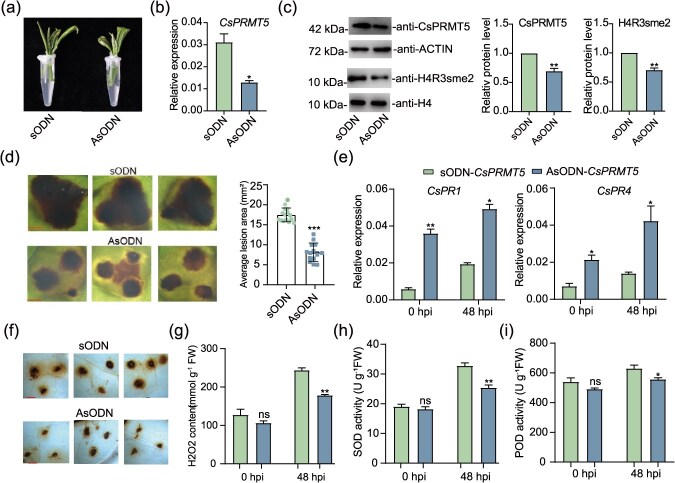
**The pathogen resistance phenotypes of *CsPRMT5*-silenced tea plants**. (a) The schematic diagram of AsODN gene silencing. (b) The relative expression levels of *CsPRMT5*. (c) Immunoblot analysis of levels of CsPRMT5 and H4R3sme2 in AsODN and sODN plants. (d) Disease symptoms after *Ps* infection in *CsPRMT5*-silenced tea plants and control. Scale bars = 0.1 mm. (e) The relative expression levels of *PR* genes. (f) Diaminobenzidine (DAB) staining, (g) H_2_O_2_ content, (h) CAT activity, and (i) POD activity of tea plant leaves 48 h after *Ps* inoculation.

To further investigate the role of CsPRMT5 in *Ps* resistance in tea plants, we transiently suppressed its expression in tea leaves using a gene-specific antisense oligonucleotide (AsODN) approach, as previously described. The new shoots of tea plants were treated by asODN and sense oligonucleotide (sODN) (Fig. 3a). RT-qPCR and immunoblot assays revealed a significant decrease in both CsPRMT5 transcript and protein levels in AsODN-treated tea plants compared to the control group treated with sODN. This reduction was accompanied by a notable decrease in H4R3sme2 levels (Fig. 3b and c). Additionally, we observed that the lesion area in the *CsPRMT5*-silenced plants was smaller than the control after 5 dpi (Fig. 3d). Pathogenesis-related (PR) genes, which play a key role in the immune response of tea plants, were upregulated early during *Ps* infection in both AsODN- and sODN-treated plants. However, *PR* gene expression was significantly higher in *CsPRMT5*-silenced plants than in the control (Fig. 3e).

Subsequently, we overexpressed *CsPRMT5* driven by the 35S promoter in tea leaves via a transient expression method. RT-qPCR analysis demonstrated a significant upregulation of *CsPRMT5* expression. Compared to the control plants that expressed the empty vector (EV), *CsPRMT5*-overexpressing tea plants had increased lesion areas and reduced resistance to *Ps*, corroborating the previously described findings (Fig. S1). Reactive oxygen species (ROS) accumulation and scavenging are common defense responses of plants against pathogen infection. We measured the activity of key ROS scavenging enzymes (Fig. 3f and i). Consistent with the disease resistance phenotypes, compared to sODN plants, H_2_O_2_ levels were lower in *CsPRMT5*-silenced plants, and the activities of POD and SOD enzymes were higher. In contrast, ROS accumulation was significantly higher in *CsPRMT5*-overexpressing leaves compared to EV (Fig. S1). These findings indicate that CsPRMT5 acts as a negative regulator of gray blight resistance in tea plants.

### The overexpression of *CsPRMT5* reduced disease resistance in transgenic Arabidopsis

**Figure 4:**
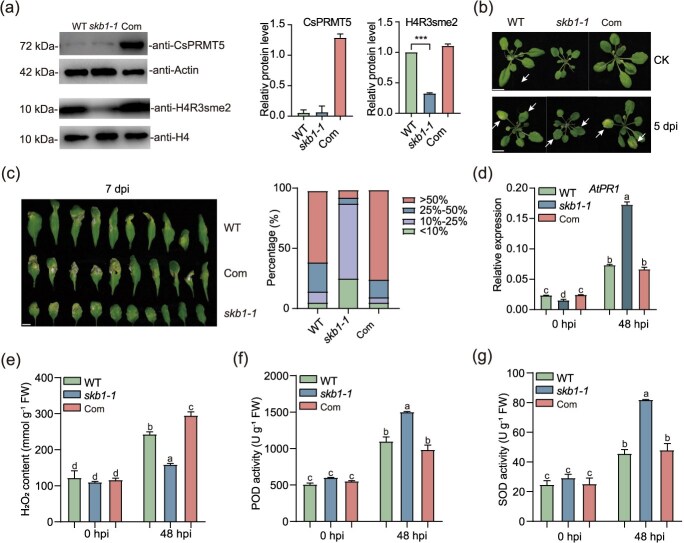
**Heterologous expression of *CsPRMT5* reduced disease resistance in transgenic Arabidopsis**. (a) CsPRMT5 and H4R3sme2 levels of WT, *skb1-1*, Com2 Arabidopsis plants. (b) Phenotypes of 5-week-old Arabidopsis WT, *skb1-1*, and Com2 plants with disease symptoms after *Ps* infection. The CK treatment (mock) was inoculated with water. Photographs were taken at 5 dpi. (c) Representative leaves with fungal infection symptoms of WT, *skb1-1*, and Com2 plants and the statistics of different grades of disease symptoms after *Ps* infection. (d) Relative expression levels of *AtPR1*, (e) H_2_O_2_ content, (f) POD activity, and (g) SOD activity. Scale bars = 1 cm.

To investigate the function of *CsPRMT5* on disease resistance in other plants, we overexpressed *CsPRMT5* in the Arabidopsis *skb1* mutant (*35S::CsPRMT5/skb1-1*, hereafter as Com2) and challenged wild-type (WT), *skb1-1* mutant, and Com2 plants with *Ps* strain. The *skb1-1* mutant plants showed significantly enhanced resistance to *Ps* infection, while the Com2 plants were more susceptible (Fig. 4b). Disease severity was categorized into four levels based on the percentage of the green area lost in half leaves. Statistical analysis indicated that Com2 and WT plants exhibited a greater proportion of Level 4 severity and a diminished proportion of Level 1 severity, whereas the *skb1-1* mutant had the inverse trend, with an increased proportion of Level 1 severity and a reduced proportion of Level 4 severity (Fig. 4c). Consistent with the phenotypic results, *AtPR1* was significantly induced in Com2 plants (Fig. 4d). Moreover, we observed stronger H_2_O_2_ accumulation and lower SOD and POD activity in Com2 leaves, whereas H_2_O_2_ levels were almost unchanged in *skb1* (Fig. 4e and g). Our results show that *CsPRMT5* negatively regulates plant resistance to gray blight.

### Pathogen-responsive genes are upregulated in *CsPRMT5*-silenced plants

**Figure 5:**
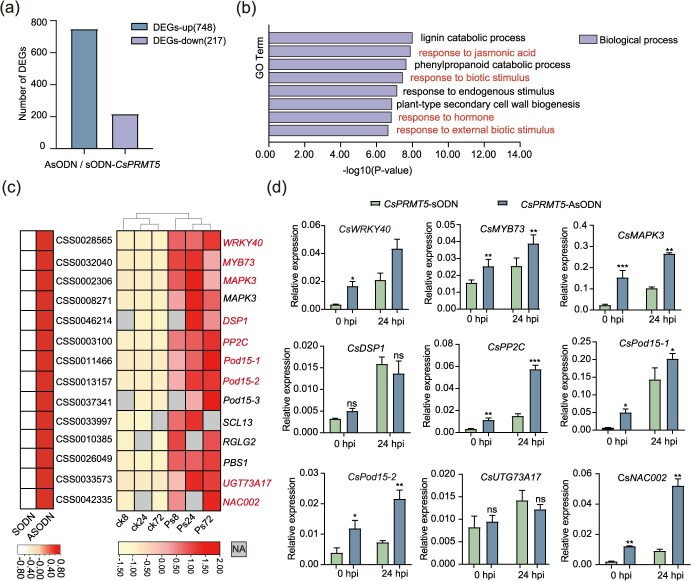
**CsPRMT5 regulates the expression of immune-related genes**. (a) Numbers of DEGs in *CsPRMT5*-silenced plants compared to sODN plants. Tea plant samples with efficient CsPRMT5 silencing were subjected to transcriptome analysis through RNA sequencing. (b) GO enrichment analysis of upregulated DEGs in *CsPRMT5*-silenced plants relative to sODN plants. (c) A heatmap showing normalized expression levels of pathogen-related genes in tea leaves following different periods of *Ps* infection. TPM (log_2_) of genes as *z*-scores. (d) The relative expression of potential CsPRMT5 target genes.

To clarify the molecular processes by which CsPRMT5 regulates tea plant immunity, we performed transcriptome sequencing to identify pathogen-associated genes governed by CsPRMT5. In *CsPRMT5*-silenced plants, a total of 748 upregulated genes and 217 downregulated genes were identified, with a log₂ (fold-change) ≥1.0 serving as the cut-off under normal growth conditions (Fig. 5a). Since CsPRMT5-mediated H4R3sme2 is generally associated with gene repression, the transcription of its targeted genes is anticipated to be stimulated in *CsPRMT5*-silenced plants. Therefore, we concentrated on the upregulated genes (Table S2). Among these genes, 48 were categorized into ‘response to biotic stimulus’ in Gene Ontology (GO) analysis (Fig. 5b; Table S4). This group includes 14 genes that were previously reported to be associated with host defense and disease resistance, such as WRKY, receptor-like kinases, and ankyrin repeat proteins; the transcriptomic data used for the heatmap are sourced from Tan *et al*. (Fig. 5c; Table S3) [6].

The expression of pathogen-responsive genes, including *CsMAPK3*, *CsPP2C*, and *CsDSP1*, was significantly induced following pathogen infection [37, 38], and their transcript levels were higher in *CsPRMT5*-silenced plants compared with sODN-treated plants, which is consistent with the RNA-seq analysis (Fig. 5d). These results suggest that CsPRMT5 negatively modulates the transcription of pathogen-induced genes.

### CsPRMT5 modulates resistance to gray blight by manipulating *CsMAPK3* expression

**Figure 6:**
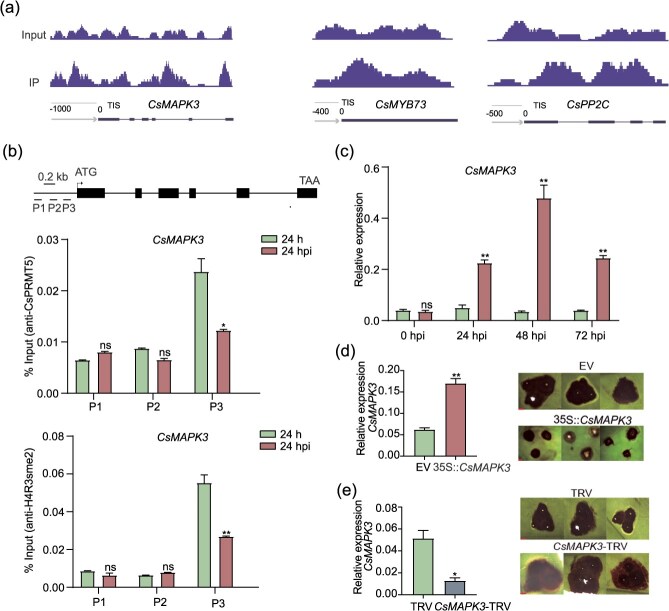
**The binding of CsPRMT5 to the chromatin of resistance genes and the levels of H4R3sme2 decreased by *Ps* infection in tea plants**. (a) ChIP-seq analysis of CsPRMT5 association with chromatin containing defense-related genes (The experiment used anti-H4R3sme2 antibodies). (b) *CsMAPK3* gene structure. Chromatin extracted from tea leaves was treated with *Ps* for 24 h. (c) The expression of *CsMAPK3* in leaves infected relative to the double-distilled water control (CK). Template RNA isolated from tea leaves was treated with *Ps* for 24, 48, or 72 h. (d) Disease symptoms following *Ps* infection in *CsMAPK3*-overexpressing and control. Scale bars = 2 mm. (e) Disease symptoms following *Ps* infection in *CsMAPK3*-silenced tea plants (CsMAPK3-TRV) and control (TRV) plants.

In Arabidopsis, SKB1 associates with the *FLC* promoter and catalyzes H4R3sme2, leading to transcriptional suppression [27, 32]. To investigate whether the elevated expression of defense genes was due to decreased H4R3sme2 levels, we first conducted a whole-genome ChIP-seq analysis using anti-H4R3sme2 antibodies (Table S4). We found that there were methylation modifications in the chromosome where pathogen-responsive genes, including *CsMAPK3*, *CsPP2C*, and *CsMYB73*, were located (Fig. 6a). We verified the binding of CsPRMT5 and H4R3sme2 in the promoters of *CsMAPK3* using ChIP-qPCR/ChIP-PCR (Chromatin Immunoprecipitation PCR). Consistently, CsPRMT5 binding was significantly enriched near or at the promoter regions (Fig. 6b and Fig. S2). Upon pathogen infection, the degree of chromatin binding of CsPRMT5 and the methylation level of *MAPK3* both decreased, while the transcription level of *CsMAPK3* increased (Fig. 6b and c). In addition, tea leaves with overexpressed *CsMAPK3* showed smaller lesion areas than that of the EV leaves, while *CsMAPK3*-silencing led to larger lesion areas (Fig. 6d and e).

**Figure 7:**
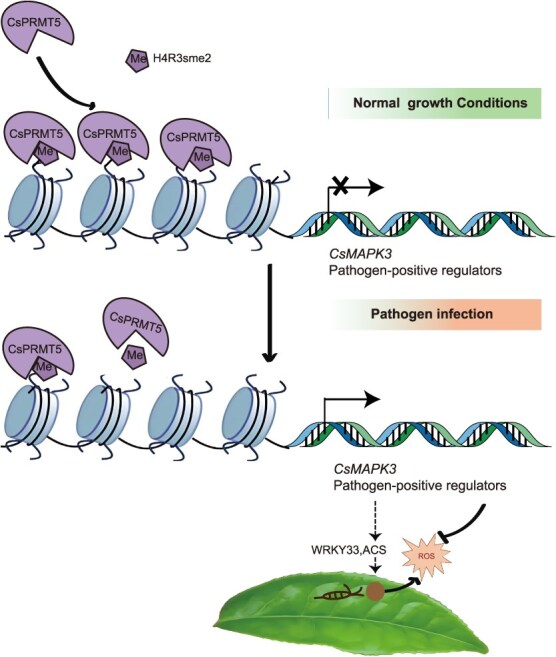
**A model of CsPRMT5 regulation of *CsMAPK3* expression represses resistance to gray blight in tea plant**. CsPRMT5 binds to defense-related genes, including *CsMAPK3*, and regulates their expression through H4R3sme2 modification. Under normal growth conditions, CsPRMT5 associates with CsMAPK3 to mediate its H4R3sme2, inhibiting the expression of CsMAPK3. After infection by the gray blight pathogen (*Ps*), the expression of CsPRMT5 is suppressed, leading to less CsPRMT5 association with chromatin and a decline in H4R3sme2 levels, thereby releasing the transcriptional suppression of pathogen-induced CsMAPK3 and enhancing plant resistance.

Building on these findings, we propose a hypothetical model outlining the role of CsPRMT5 in the tea plant defense response (Fig. 7). In the pathogen infection, CsPRMT5 binds to chromatin to inhibit the expression of disease-resistance genes through arginine methylation to maintain normal plant growth. In contrast, when tea plants are infected by fungi, the expression of *CsPRMT5* decreases. Consequently, less CsPRMT5 associates with the chromatin and H4R3sme2 level declines, thereby releasing the transcriptional suppression of pathogen-responsive genes and increasing the activity of ROS scavenging enzymes, which plays a positive role in the disease resistance of tea plants.

## Discussion

In this study, we propose a mechanism by which CsPRMT5-mediated histone H4R3 dimethylation negatively regulates resistance to gray blight in tea plants (Fig. 7). As an important mechanism of epigenetic regulation, histone modifications (such as acetylation and lysine methylation) have been widely reported implicating in a variety of biological processes [39], while the specific role of histone arginine methylation in plant immunity remains unclear. In this study, we showed that CsPRMT5 regulates plant disease defense epigenetically through the H4R3sme2 in tea plants. The levels of H4R3sme2 are closely associated with the external environment. In *CsPRMT5*-silenced tea plants and *skb1-1* mutant, genes involved in plant immune responses were upregulated and the absence of H4R3sme2 enhanced plant resistance to *Ps* infection (Fig. 5). Our findings strongly suggest that CsPRMT5 contributes to the defense against gray blight through the modulation of resistance-related genes in plants.

Histone methylation plays a fundamental role in regulating various developmental processes and is also involved in silencing repetitive sequences to maintain genome stability [17]. Pathogen-induced histone methylation modifications can activate host cell signaling cascades, activating or inhibiting the expression of defense genes, thereby enhancing or suppressing plant resistance responses. The most extensively studied modifications are the methylation/demethylation of histone lysine residues, with modifications at the H3K4, H3K27, and H3K36 sites upregulating SA/JA signaling pathway genes or R genes, positively regulating plant resistance to *Pst DC3000* and *Xoo* [40]. In contrast, the regulatory mechanisms of histone arginine methylation in plant immunity remain to be further explored. This study finds that histone arginine methylation exhibits dynamic changes during pathogen infection. The repressive mark H4R3sme2 mediated by PRMT5 significantly decreases upon pathogen infection, which is consistent with previous reports in potatoes [41].

It is noteworthy that PRMT5, as a multifunctional regulatory factor, regulates flowering time by inhibiting FLOWERING LOCUS C (FLC) gene expression through H4R3sme2 and is also involved in salt stress response [32]. During salt stress or ABA treatment, PRMT5/SKB1 dissociates from chromatin, leading to a decrease in H4R3sme2 levels, which activates FLC and stress-responsive genes and enhances pre-mRNA splicing efficiency. Vernalization or photoperiod treatment dynamically alters the H4R3sme2 levels on the FLC chromatin to regulate the flowering process. Additionally, PRMT5/SKB1-mediated H4R3sme2 deposition in the promoter region of the Ib subgroup bHLH transcription factors alleviates gene repression under iron deficiency conditions by reducing the modification levels, enhancing Arabidopsis iron uptake ability [42]. Together, these findings suggest that PRMT5 participates in plant development and environmental adaptation by dynamically regulating H4R3sme2.

This study further reveals the novel function of CsPRMT5 in plant immunity. Transcriptome analysis showed that differentially expressed genes in *CsPRMT5*-silenced tea plants are significantly enriched in the plant–pathogen interaction pathway, which is highly consistent with the transcriptome of the Arabidopsis *skb1-1* mutant (Fig. S3; Table S6). Key immune regulators *MAPK3*, *PP2C*, *DSP1*, *WRKY40*, and *POD* are significantly upregulated in the silenced lines, and both *CsPRMT5*-silenced tea plants and Arabidopsis *prmt5* mutants exhibit enhanced disease resistance. More importantly, this study provides the first evidence that CsPRMT5 regulates plant resistance through the modulation of ROS balance. In the *CsPRMT5*-silenced plants, the expression of POD genes is significantly upregulated, resulting in a decrease in H_2_O_2_ and O^2−^ levels, which effectively mitigates oxidative damage caused by pathogen infection. In contrast, the *CsPRMT5*-overexpressing plants accumulate excessive ROS, leading to increased tissue damage and reduced resistance. These findings not only confirm that CsPRMT5 is involved in regulating gray blight disease resistance in tea plants but also reveal its role in precisely regulating defense responses and oxidative damage by coordinating ROS dynamic balance.

PRMT5 participates in gene expression regulation, RNA processing, pre-mRNA splicing, and protein homeostasis maintenance through the extensive methylation of histone and nonhistone substrates. Previous studies have revealed that PRMT5 regulates plant immunity by methylating nonhistone proteins such as AGO2 and LSM4. In Arabidopsis, PRMT5 regulates the selective splicing function of the splicing body component LSM4 through arginine methylation. Bacterial infection reduces LSM4 methylation levels, enhancing plant resistance, a mechanism related to reduced intron retention of immune-related genes. Moreover, PRMT5-mediated AGO2 methylation has been shown to finely regulate pathogen defense responses in both rice and Arabidopsis [33, 35]. This study elucidates that PRMT5 participates in tea plant immune regulation through H4R3sme2 modification. Notably, under pathogen infection conditions, the reduced binding ability of CsPRMT5 to chromatin leads to a decrease in H4R3sme2 levels, which subsequently activates the transcription of immune-regulatory genes such as *MAPK3*. However, the molecular sensing mechanism by which CsPRMT5 perceives pathogen signals and dynamically regulates H4R3 methylation remains to be further elucidated.

Previous studies have confirmed that PRMT5 (SKB1) is crucial for plant growth and development. Compared to WT plants, the Arabidopsis *skb1* mutant exhibits traits such as late flowering, darker leaves, and slower growth rates. In tea plants, PRMT5 shows different expression patterns in various tissues, with significantly higher expression in young leaves (Fig. S4). However, the specific functions of PRMT5 in different developmental stages of tea plants (such as germination and flowering) remain largely unexplored and warrant further investigation. Studies on the Arabidopsis *skb1-1* mutant show that the loss of PRMT5 leads to growth inhibition but enhances disease resistance, revealing its pivotal role in coordinating resource allocation. PRMT5 binds to chromatin and deposits the repressive marker H4R3sme2, directly suppressing the expression of the flowering regulator gene FLC. Pathogen infection triggers a decrease in PRMT5 protein levels, which in turn reduces the methylation modification of the spliceosome component LSM4, enhancing resistance by optimizing the alternative splicing of immune-related genes. This bidirectional dynamic modification suggests that PRMT5 may function as a ‘molecular switch’ to balance growth and immune metabolic conflicts through epigenetic reprogramming.

This study reveals a new mechanism by which CsPRMT5 negatively regulates H4R3sme2 levels to suppress disease resistance in tea plants, providing an epigenetic target for crop disease resistance breeding. Future research could focus on: elucidating how CsPRMT5 collaborates with DNA methylation/histone acetylation to coregulate the defense gene network; clarifying the functional division and cross-regulation between CsPRMT5 and other PRMT members in tea plants (such as CsPRMT3, CsPRMT10); exploring the conservation and specificity of PRMT5-mediated disease resistance pathways in economic crops like *Solanum lycopersicum* and *Zea mays*, and assessing their potential for broad-spectrum disease resistance.

## Materials and methods

### Plant materials and growth conditions

This study used the ‘Shuchazao’ cultivar as the primary experimental material, which was cultivated in the Experimental Tea Garden of Anhui Agricultural University (latitude 31.86°N, longitude 117.27°E). To test disease resistance of different cultivars, seven tea plant cultivars, including ‘Longjing 43’ (LJ43), ‘Shuchazao’ (SCZ), ‘Wuniuzao’ (WNZ), ‘Fuyun 6’ (FY6), ‘Longjingchangye’ (LJCY), ‘Zhenong 113’ (ZN113), and ‘Pingyangtezao’ (PYTZ). *Arabidopsis thaliana* (Columbia-0, Col-0), mutant *skb1-1* (salk_065814), and the complementary line (35S::*CsPRMT5 skb1-1*) previously obtained by the lab were used to study the resistance against *Ps.*

### Pathogen inoculation

The *Ps* strain used is named EC-4, and its pathogenicity was reported by Wang *et al* [5]. The strain of *Ps* a was cultured on potato dextrose agar (PDA) medium at 28°C for 5 days, and then the spores were collected by centrifugation at 6000 × g for 10 min. The spore concentration was adjusted to 10^6^/ml with sterile water for subsequent inoculation tests. A sterile needle was used to create wounds on each leaf, and 50 μl of the spore suspension was inoculated onto the tea leaves. The control group plants were inoculated with the same amount of sterile distilled water. After inoculation, the leaves were covered with a thin film to maintain high humidity and promote fungal growth. The film was removed 24 h later, and regular cultivation continued. Arabidopsis was grown in nutrient soil at 22°C, with 75% humidity and a 16 h/8 h (light/darkness) photoperiod. A 15 μl spore suspension of the same concentration was applied to 5-week-old leaves of transgenic Arabidopsis*,* and with 80% humidity. The disease progression was observed in the later stages, and the lesion area was quantified using ImageJ software.

### Transient overexpression and gene silencing in tea leaves

Transient gene expression and silencing were conducted following a previously established protocol [8]. The complete cDNAs of the target genes were incorporated into the expression vector pCAMBIA1305, which contains the GFP tag, to produce the donor plasmids [43]. The primers are included in Table S5. The donor plasmids were integrated into the *Agrobacterium tumefaciens* GV3101 (pSoup-p19). Leaf samples were collected 48 hpi for further gene expression profiling and evaluation of disease resistance.

AsODNs directed against *CsPRMT*5 were formulated utilizing Soligo software [43], with sODNs employed as controls (Table S1). Tender shoots were selected for the AsODN experiment and submerged in 100 μM AsODN/sODN; after 48 h, the leaves were collected [8]; the VIGS method was utilized to mute CsMAPK3. The 54- to 327-bp fragments from the 5′ regions of CsMAPK3 were employed as silencing pieces.

### Immunoblot analysis

Western blot examination of total proteins was conducted with anti-CsPRMT5 polyclonal antibodies (Wuhan Aibo Tech; projected molecular weight, 72 kDa). Histone-enriched proteins were obtained following the methodology outlined by Houben *et al*. [44], and immunoreacted samples, as detailed by Connolly *et al*., were utilized to assess CsPRMT5 levels [45]. Immunoblot analysis was conducted using antibodies against Anti-Histone H4/Anti-H4R3sme2 (Shanghai Aibo Antibody; anticipated molecular mass, 10/15 kDa). ACTIN served as the loading control (Shanghai Bioengineering; anticipated molecular weight, 42 kDa). The original images of the immunoblot analysis have been added to Table S7.

### Histochemical staining of plants and assessment of reactive oxygen species

The leaves infected with the pathogen were positioned in 3-methylene-diaminobenzidine (DAB) solution (1 mg/ml, pH 3.8) and incubated at 37°C for 8 h. The enzyme activity of SOD and POD in the leaves were measured using kits (Beijing Solabao Technology Co., Beijing, China) [46].

### ChIP-qPCR assay

We conducted ChIP analysis as previously described with minor modifications [47]. Samples infected by *Ps* for 24 h were used for the ChIP-qPCR assay. The samples were placed in a 50-ml centrifuge tube, and 1% formaldehyde was added for cross-linking under vacuum conditions for 30 min. Then, 0.125 mol/l glycine was added to terminate the cross-linking. The plant material was pulverized in liquid nitrogen, and the nuclei were extracted. The chromatin was then fragmented using sonication. DNA fragments bound to proteins were immunoprecipitated with anti-H4R3sme2 (Shanghai Aibo Antibody, 1:5000 dilution). The immunoprecipitated DNA fragments were extracted and purified for ChIP-qPCR assays. The percentage input was calculated by 1% × 2 ^(Ct)1%Input Sample-(Ct)IP Sample^_._ The dilution factor (IDF) was 100, and the Input DNA was 1%.

### RNA-seq analysis

Total RNA was extracted using the RNA Pure Plant Kit (Tiangen). Subsequently, the quality and concentration of the extract were assessed using agarose gel electrophoresis and the NanoDrop 2000 spectrophotometer (Thermo). The library was then constructed and sequenced using the Illumina sequencing platform. After filtering out low-quality reads, all remaining high-quality clean sequencing reads were aligned to the tea plant reference genome (http://tpdb.shengxin.ren/). AsODN- and sODN-treated samples from one bud and one leaf were extracted and sent to Novogene Technology Co., Ltd. for sequencing. Each sample included three biological replicates. Differentially expressed genes (DEGs) in response to AsODN treatment were defined using a threshold of fold change ≥1.00 and *P* ≤ 0.05. The data of gray blight are sourced from the public database of Anhui Agricultural University (http://tpia.teaplant.org/index.html).

### Statistical analysis

All studies were performed with at least three distinct biological replicates. All measurements were performed in duplicate. A one-way analysis of variance (ANOVA) was utilized to evaluate statistically significant changes between the control and experimental plants, employing Duncan’s test (*P* < 0.05) executed through SPSS.

## Supplementary Material

Web_Material_uhaf100

## Data Availability

The data presented in this study are available on request from the corresponding author. The raw transcriptome data have been deposited in the National Center for Biotechnology Information bioproject database under the accession number PRJNA1223124 for the RNA-seq (CsPRMT5 suppression in tea plants) and PRJNA1223117 for the ChIP-seq data, respectively.
