# Supplementary material for: CsPRMT5-mediated histone H4R3 dimethylation negatively regulates resistance to gray blight in tea plants (Camellia sinensis L.)
Source: Hortic Res. 2025 Apr 9;12(7):uhaf100. doi: 10.1093/hr/uhaf100 (PMC12090350; doi:10.1093/hr/uhaf100)
Supplement: Web_Material_uhaf100 [file web_material_uhaf100.zip › Supporting Figures.docx]

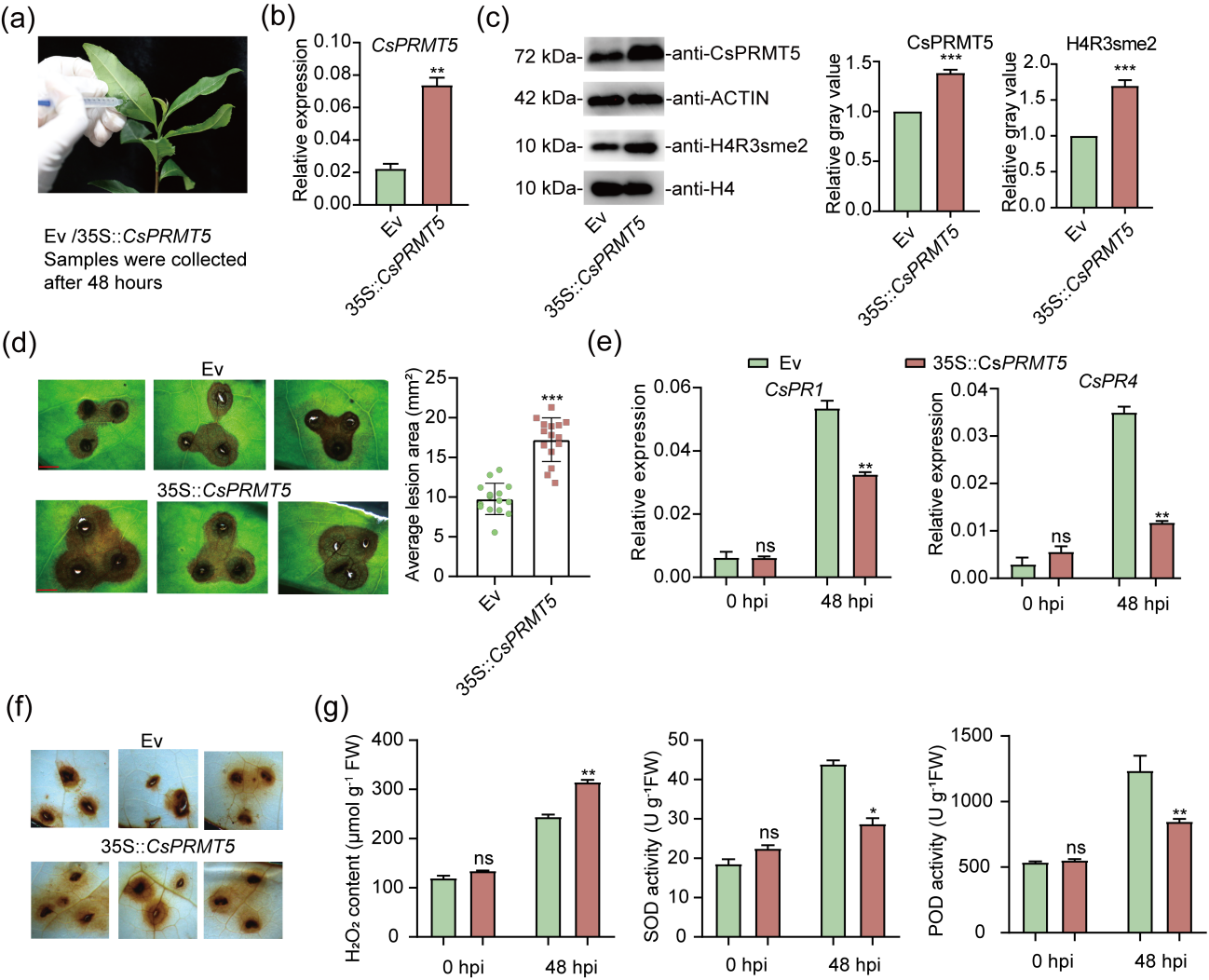
Figure S1. **The pathogen resistance phenotypes of *CsPRMT5*-overexpressing tea plants.** (a) Schematic diagram of transient genetic overexpression. (b) The relative expression levels of *CsPRMT5* (c) Immunoblot analysis of levels of CsPRMT5 and H4R3sme2 (d) Disease symptoms after fungal infection. Scale bars = 0.1 mm. (e) The relative expression levels of *PR* genes in *CsPRMT5*-overexpressing and control (EV) tea plants. (f) Diaminobenzidine (DAB) staining, (g) H_2_O_2_ content, CAT and POD activity of tea plant leaves 48 h after *Ps* inoculation.


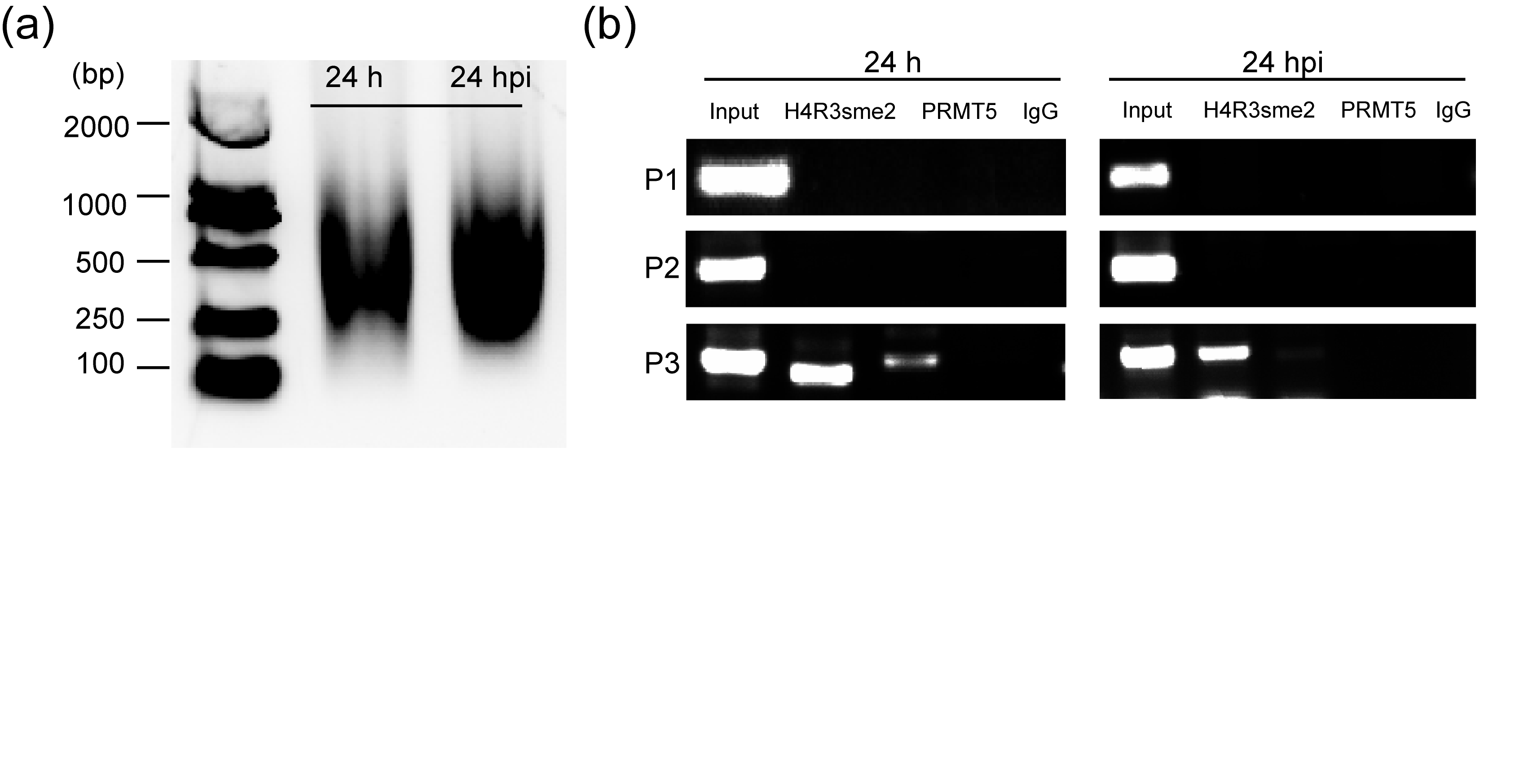

Figure S2. **ChIP-PCR.** (a) The chromatin fragments were fragmented to 250-500 bp. (b) The binding of CsPRMT5 to the chromatin of CsMAPK3.


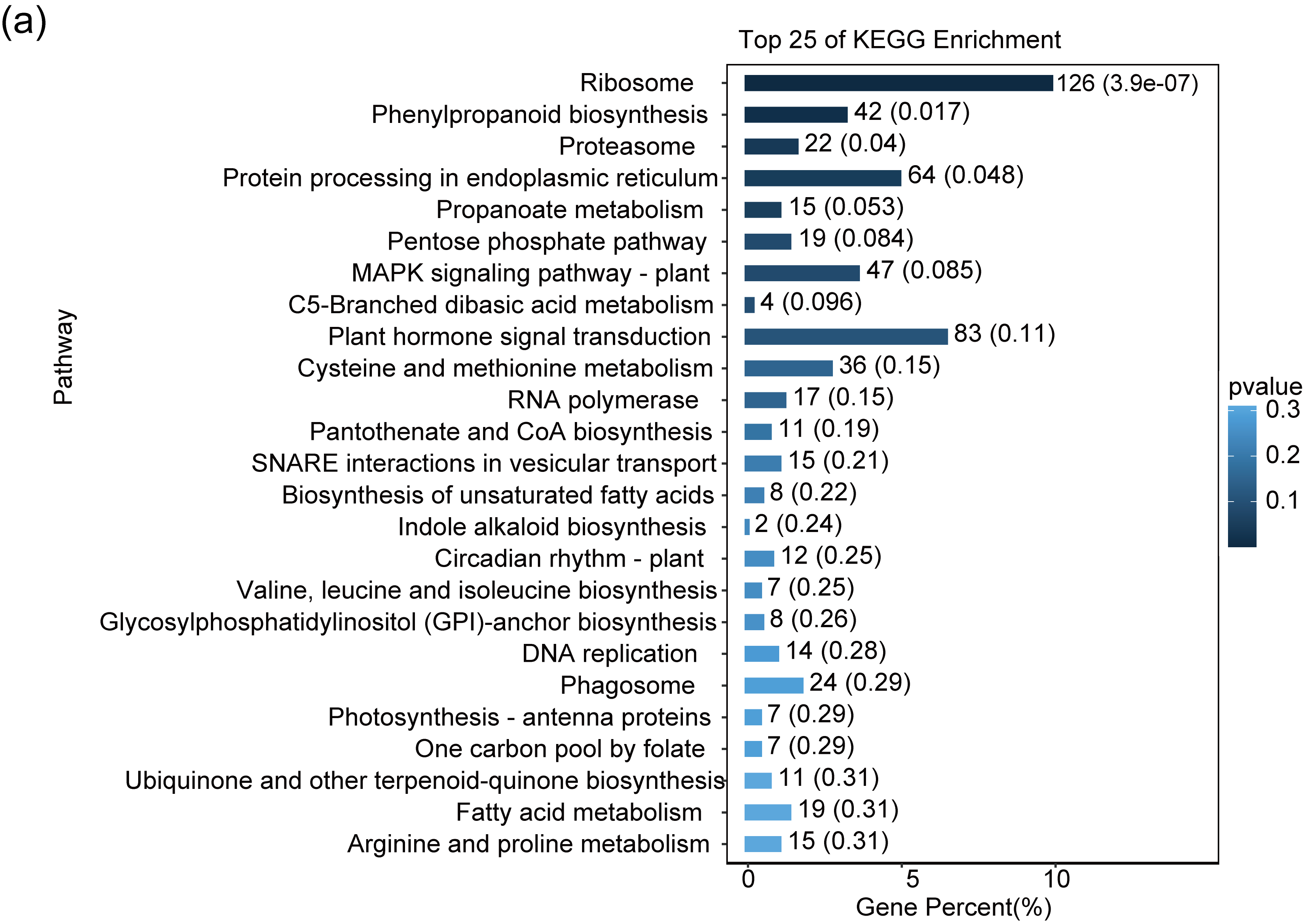


Figure S3. **KEGG enrichment analysis.** (a) KEGG enrichment analysis of DEGs in KEGG enrichment analysis of DEGs in n *skb1-1* vs Col-0 .


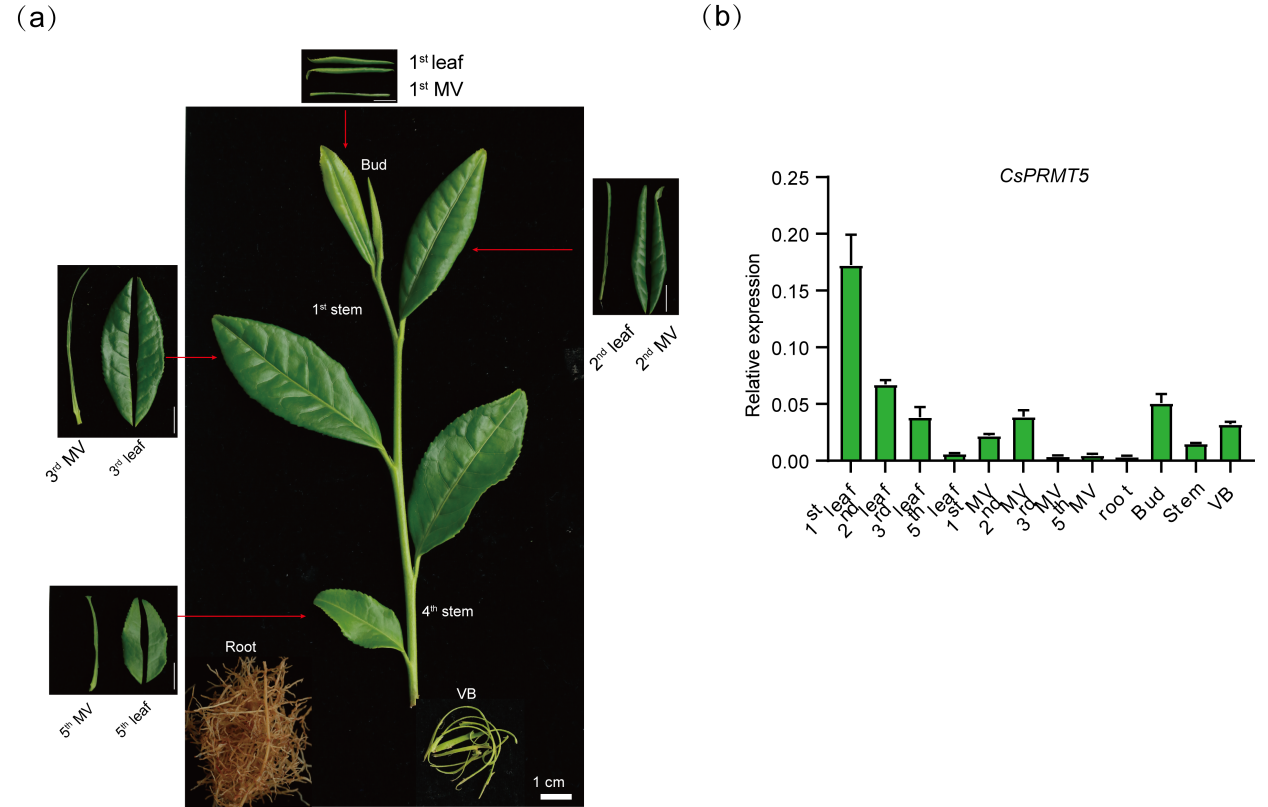


Figure S4. **Expression of *CsPRMT5* in different tissues of tea plant.**
